# Label-Free Optical Sensing and Medical Grade Resins: An Advanced Approach to Investigate Cell–Material Interaction and Biocompatibility

**DOI:** 10.3390/pharmaceutics15082043

**Published:** 2023-07-29

**Authors:** Valentina Bergamini, Elisa Resca, Alberto Portone, Tiziana Petrachi, Francesco Ganzerli, Stefano Truzzi, Giorgio Mari, Luigi Rovati, Massimo Dominici, Elena Veronesi

**Affiliations:** 1 Clinical and Experimental Medicine PhD Program, University of Modena and Reggio Emilia, 41125 Modena, Italy; valentina.bergamini@tpm.bio; 2 Technopole “Mario Veronesi”, Via 29 Maggio, 41037 Mirandola, Italy; elisa.resca@tpm.bio (E.R.); alberto.portone@tpm.bio (A.P.); tiziana.petrachi@tpm.bio (T.P.); francesco.ganzerli@tpm.bio (F.G.); truzzi.stefano@gmail.com (S.T.); giorgio.mari@tpm.bio (G.M.); luigi.rovati@unimore.it (L.R.); massimo.dominici@unimore.it (M.D.); 3 Department of Engineering “Enzo Ferrari”, University of Modena and Reggio Emilia, Via Vivarelli, 10, 41125 Modena, Italy; 4 Department of Medical and Surgical Sciences for Children & Adults, University of Modena and Reggio Emilia, Hospital of Modena, Via del Pozzo, 71, 41125 Modena, Italy

**Keywords:** label-free technology, biocompatibility, medical device

## Abstract

The Corning Epic^®^ label-free (ELF) system is an innovative technology widely used in drug discovery, immunotherapy, G-protein-associated studies, and biocompatibility tests. Here, we challenge the use of ELF to further investigate the biocompatibility of resins used in manufacturing of blood filters, a category of medical devices representing life-saving therapies for the increasing number of patients with kidney failure. The biocompatibility assays were carried out by developing a cell model aimed at mimicking the clinical use of the blood filters and complementing the existing cytotoxicity assay requested by ISO10993-5. Experiments were performed by putting fibroblasts in both direct contact with two types of selected resins, and indirect contact by means of homemade customized well inserts that were precisely designed and developed for this technology. For both types of contact, fibroblasts were cultured in medium and human plasma. ELF tests confirmed the biocompatibility of both resins, highlighting a statistically significant different biological behavior of a polyaromatic resin compared to control and ion-exchanged resin, when materials were in indirect contact and soaking with plasma. Overall, the ELF test is able to mimic clinical scenarios and represents a promising approach to investigate biocompatibility, showing peculiar biological behaviors and suggesting the activation of specific intracellular pathways.

## 1. Introduction

Over 2 million people worldwide suffer from kidney failure, and the number of patients continues to increase by 5–7% every year. The only life-saving therapy, other than organ transplantation, is dialysis, where blood filters are used. Blood filters are designed and manufactured to remove waste products from blood in patients with kidney failure. Like all medical devices (MDs), they are subject to extensive and rigorous in vitro and in vivo testing to ensure their biocompatibility and suitability for treating patients. Although testing methodologies are standardized and regulated, numerous innovative techniques able to enhance the efficiency of biocompatibility evaluation are emerging, potentially revolutionizing the field. Among these, label-free methods offer various advantages over traditional approaches, such as eliminating the need for cell labeling, simplifying assay design, and reducing the risk of artifacts caused by labeling processes. Additionally, noninvasive measurement allows for both real-time kinetic and endpoint measurements, thereby enabling a wide range of studies [1,2].

**Figure 1:**
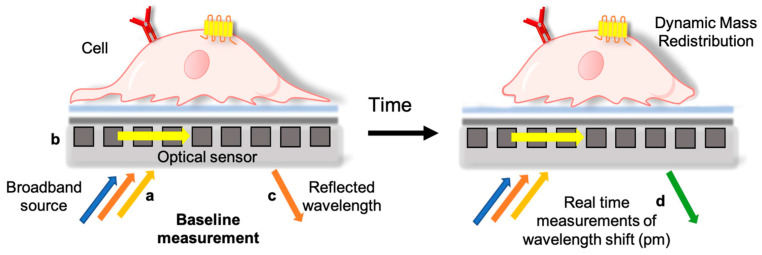
Representative depiction of a cell in contact with the sensors of the label-free plate. A resonant waveguide grating biosensor is placed on the bottom of each well of the Enspire multiplate (Perkin Elmer, Waltham, MA, USA), which is able to measure changes in the local index of refraction and detect the DMR present within the bottom region (~150 nm) of the cell monolayer. When the plate is irradiated by a beam of broadband white light (a), light is diffracted and guided along the sensor grid (b), and a part is reflected with an angle proportional to the incident beam (c). The peak position of the reflected wavelength spectrum is detected and analyzed using a spectrometer within the label-free technology module (d). The instrument carries out a baseline measurement at time 0, which evaluates the starting situation of the adhered cells. After the addition of the test compound, the instrument takes measurements over time, calculating the shift of the maximum peak of the reflected light (named “response” and expressed in pm) due to the DMR, which provides real-time information on changes in cellular behavior.

In this framework, one of the most promising technologies is the Corning Epic^®^ label-free (ELF) system [2]. This is a highly sensitive biosensing method that utilizes a broadband light source, an optical detector, and specific micro-plates embedding a Resonance Waveguide Grating (RWG) sensor in the bottom of each well. The RWG is a substrate with a diffractive optical grating and a waveguide coating. Coupling of a broadband light with the waveguide layer generates an evanescent wave that interacts with the surface for a penetration depth of about 150 nm. Only a narrow band of wavelengths is reflected from the surface and its peak depends on the refractive index of the density and biomass distribution (called dynamic mass redistribution, DMR) of the structures that the evanescent waves encounter on the surface [2]. If cells are seeded on the RWG surface (Figure 1), any changes in the steric distribution of cell mass due to migration, proliferation, cell death, or specific stimulation-mediated cell responses result in a shift in the reflected light peak, expressed in picometers (pm) [2,3]. In a typical ELF evaluation of material biocompatibility, an initial reading, named “baseline”, measures cells adhered to the plastic under physiological conditions; the reading is then repeated after adding the compound to be tested. The eventual difference in DMR determines a shift in the optical signal; an increase in the raw signal is associated, for instance, with an increase in cell mass, and it is referred to as positive-DMR (P-DMR); conversely, a decrease in response is associated with cell shrinkage, and it is reported as negative-DMR (N-DMR). The timeline of all DMR phases generates a phenotypic profile for the corresponding cell population [3]. Morphological change in the transport proteins, the rearrangement of the cytoskeleton, and the internalization of receptors are some examples of biological mechanisms causing a DMR.

Although ELF is broadly used in the pharmacology field, few studies report applications of ELF for biocompatibility studies. However, our group has previously utilized the technique to investigate the biocompatibility of methacrylate resins used in cranioplasty surgery. In that work, various cell types, including those from bone, vascular, and neural tissues, were exposed to the resins and to positive and negative controls in order to simulate the in vivo interaction of the material. The results revealed ELF’s efficacy in distinguishing between toxic and non-toxic compounds [3].

Here, we demonstrate the possibility of using ELF to investigate the biocompatibility performances of other relevant materials in the biomedical field, dedicating our attention to blood filters, with a specific focus on their two primary functional components: (i) the ion-exchange resins and (ii) the macroporous polymeric resins.

Ion-exchanged resins are present in the anion-exchange membrane (AEM) of the filter. They bind, sequestrate, and then remove target compounds from blood during dialysis treatment [4,5,6]. This kind of resin has ionizable groups that dissociate and undergo an ion exchange with the external solution, especially in an aqueous environment [7].

Macroporous polymeric resins were developed and are primarily used for adsorption and reversed-phase liquid chromatography [8]. In blood filters, their main application is to remove inflammatory mediators in the treatment of systemic inflammatory diseases that occur in patients undergoing dialysis for kidney failure. Furthermore, they are able to bind factors such as VEGF, myoglobin, and C-reactive protein, but also cytokines and chemokines (IL-1, IL-6, IL-8, IL-12, IL-18, TNF) [6].

In this biocompatibility study, we selected murine fibroblast L929 as a cell model to better compare the results obtained with the MTT (3-(4,5-dimethylthiazol-2-yl)-2,5-diphenyltetrazolium bromide) cytotoxicity assay described by ISO 10993-5, which are required for the manufacturing of blood filters [9]. Given the microenvironment and the fine balance where blood filters work, we challenged a cell model where fibroblast and materials are in co-culture, to better investigate the biological effect of resin on fibroblasts with or without the interaction of human plasma. For this purpose, a very sensitivity technology such as ELF is suitable for investigations into the role of resins in the microenvironment that mimics the clinical scenario.

## 2. Materials and Methods

### 2.1. Cells

L929s—purchased from ATCC (Manassas, VA, USA)—were thawed and amplified according to the manufacturer’s instructions using culture media composed of Dulbecco Modified Medium (DMEM) (Gibco-ThermoFisher, Waltham, MA, USA) supplemented with 10% of fetal bovine serum (FBS) (Euroclone, Pero, MI, Italy), 1% Penicillin-Streptomycin (Gibco-ThermoFisher, Waltham, MA, USA), and 1%, Glutamine (Gibco-ThermoFisher, Waltham, MA, USA).

Once 90% confluence was reached, cells were detached from the flask by trypsinization; 0.05/0.002% Trypsin-EDTA (Euroclone) was added for 3 min and then blocked with culture medium. Detached cells were collected and centrifuged at 1400 rpm for 5 min. The resulting pellet was then resuspended and seeded at the density of 15,000 cells per cm^2^ into a microwell plate having 96 wells (Perkin Elmer).

### 2.2. Plasma

Plasma was collected by spinning at 700–800 g/15 min the human blood received by the University Hospital of Modena and Reggio Emilia (according to Decree of the Ministry of Health of 2 November 2015 reporting “Provisions relating to the quality and safety requirements of blood and blood components”). All the blood bags were negative for the mandatory infectious tests.

### 2.3. Resins

**Figure 2:**
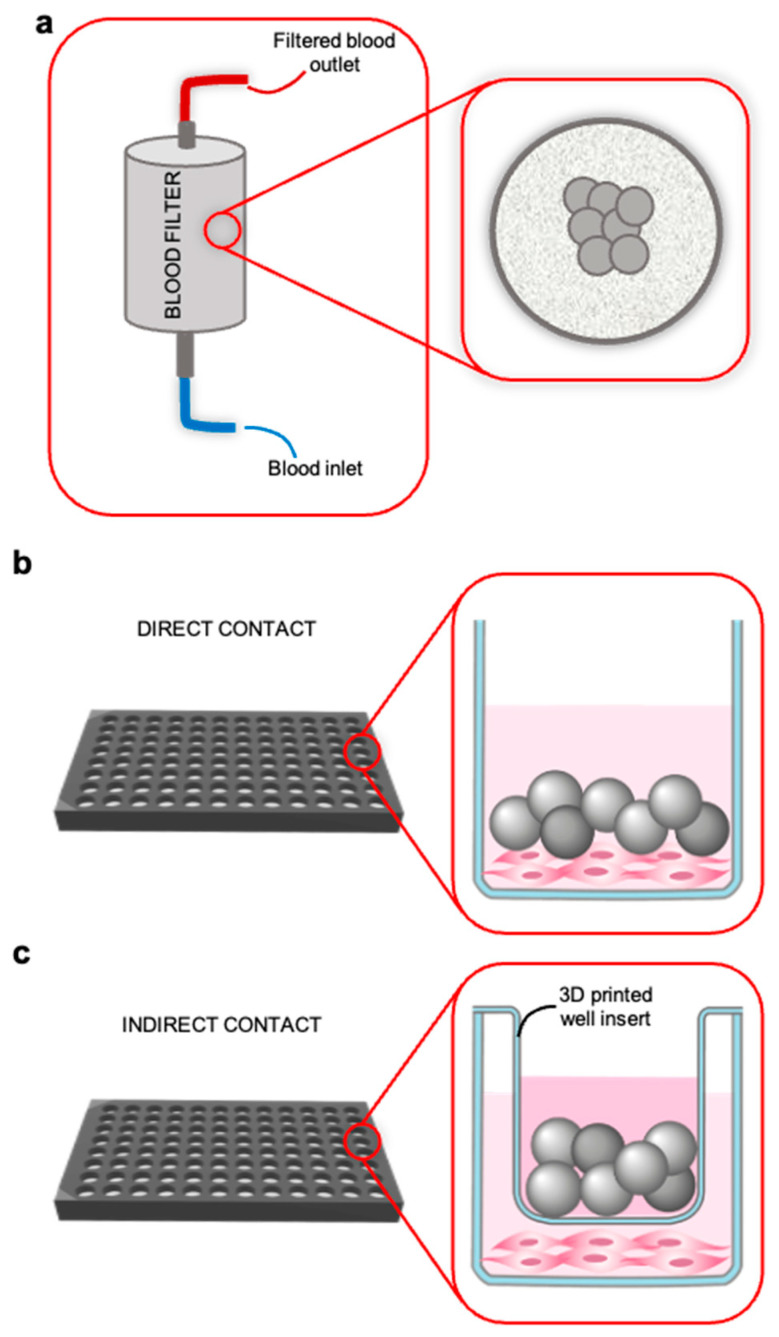
Schematic representation of the use of resins inside a blood filter of a machine for the AEM-diffusion dialysis process (**a**) and project flowchart (**b**,**c**). The two types of resins were placed inside the plate for the label-free technology in which the cells had already been previously seeded. The cellular response was analyzed following the direct contact (**b**) of the resins with the cells and following the indirect contact (**c**) by addition of a 3D-printed well insert ((**right**) panel).

Two different types of resins were tested with our ELF in vitro assay employed in blood filters (Figure 2a). The first type of resin, hence called “Resin 1”, is an ion-exchange resin and is a compound of AEM, used typically in the AEM-diffusion dialysis process. This type of resin was kindly donated by Danube University Krems, Center for Biomedical Technology, Krems, Austria [4].

The second type of resin tested in this study was a polyaromatic resin used for rapid adsorption of hydrophobic compounds such as surfactants, medium-sized proteins, polypeptides, large peptides, and antibiotics. This resin was developed and produced by Dupont^TM^ and is commercially identified as “Amberchrom CG300M”. Here, we call this resin “Resin 2” [8].

Resin samples were washed in 99.9% ethanol (Histo-Line Laboratories, Pantigliate, MI, Italy), rinsed in 0.9% saline solution Versylene^®^ NaCl (Fresenius-kabi, Bad Homburg vor der Höhe, Germany), centrifuged at 1690 g for 15 min to remove any residual alcohol, and finally sterilized under UV light for one hour.

### 2.4. Resins–Cells Co-Culture

A quantity of 5 mg of each resin was co-cultured in direct contact with fibroblasts in culture media and in human plasma, as shown in Figure 2b. As resins are not in direct contact with fibroblasts during clinical therapy, but with blood, we used a customized well insert to better simulate the physiological scenario, with an indirect contact (Figure 2c) with either culture media or plasma.

### 2.5. Development of Customized Well Insert for ELF Plate

**Figure 3:**
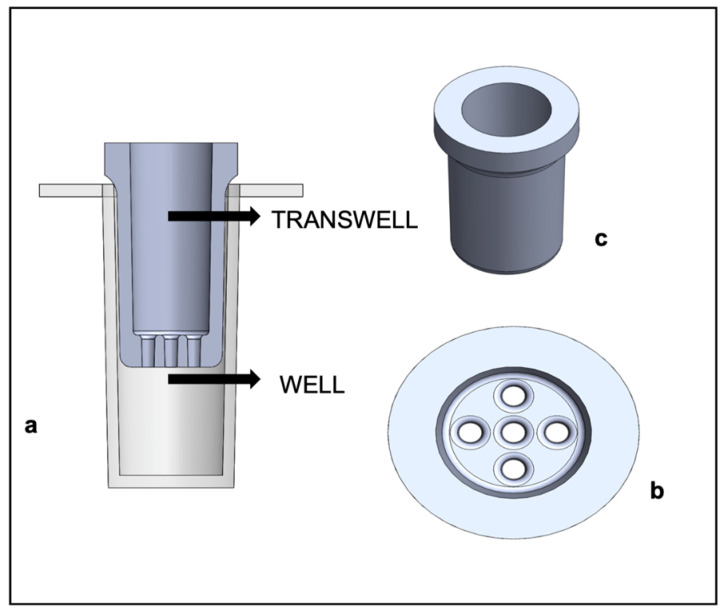
Design and printing of transwells for the label-free method. (**a**) Top view of the transwell in CAD, (**b**) three-dimensional model of the transwell in CAD, (**c**) sagittal view of the model in CAD.

To investigate the cytotoxicity effect exerted by resins alone or combined with plasma, we developed a new type of well insert that is able to be inserted into the microwell of the 96-well ELF plate (Figure 3). Resins can release and exchange ions and compounds that can affect the fibroblasts, seeded on the bottom of the microwell. In this way, resins do not directly crush cells.

Well inserts were designed using Solidworks CAD software (Solidworld 1.0.14, 2016) and realized using the commercial 3D printer Stratasys^®^-Eden 260V and a suitable photopolymerizing material, Veroblack (Stratasys^®^, Rehovot, Israel). Such inkjet technology realizes a 3D object by depositing a series of material drops onto the printing platform. The structure is built layer by layer and UV light is employed to photopolymerize the drops after every layer deposition. About 10 min elapsed to finish printing all the inserts; after that, the inserts were cleaned with compressed air and washed in water (to remove any residues of the printing material).

### 2.6. Evaluation of Cytotoxicity by 3-(4,5-Dimethylthiazol-2-yl)-2,5-diphenyltetrazolium Bromide (MTT) Assay

L929s were seeded into 96-well plates (10,000/100 µL/well) and cultured in DMEM + 10% FBS + 1% P/S, 2% Glutamine at 37 °C and 5% CO_2_. Cells were stimulated with the extracts previously obtained by incubating the resins in medium or plasma for 24 h at 37 °C, according to ISO10993-12:2021 [10] and ISO10993-5:2009 [1].

Sodium Dodecyl Sulfate (SDS, Sigma Aldrich, St. Louis, MO, USA) and 0.2% Dimethyl sulfoxide (DMSO, AL.CHI.MI.A SRL, Ponte S. Nicolò, PD, Italy) were used as positive and negative controls, respectively. After 24 h of exposure, cells were incubated with 50 µL of MTT solution (Sigma-Aldrich, St. Louis, MO, USA) for 2 h at 37 °C/5% CO_2_. A multi-plate reader spectrophotometer (Enspire, PerkinElmer, Hopkinton, MA, USA) was used to quantify the optical density at 570 nm after MTT solution remotion and the subsequent suspension of cells in 100 µL of isopropanol (Sigma-Aldrich, St. Louis, MO, USA). The reduction in cell viability compared with the negative control was determined using the following formula:

(1) $$Cell\;viability\left(\%\right)=100\times OD570e/OD570b$$

where OD570e is the mean value of the measured optical density of the 100% extract, and OD570b is the mean value of the measured optical density of the blanks. A viability greater than 70% of the blank was evaluated as not cytotoxic. Each experiment was performed six times (technical replicates).

### 2.7. Evaluation of Cytotoxicity in a Direct Cell–Resin Contact Model by ELF

Five thousand murine fibroblasts were seeded in 80 µL of culture medium for each well of the 96-well microplate and incubated at 37 °C overnight. The next day, after the culture medium had been replaced with a fresh medium, the plate was placed in an incubator for 90 min and subsequently in Enspire (Perkin Elmer), at a temperature of 37 °C for a further 30 min; then, a “baseline” measurement was acquired as 5 repetitions, 1 every 60 s. Subsequently, 70 µL of 0.1% SDS or 0.2% DMSO or 5 mg resins were added to the well. After 24 h of incubation at 37 °C and 5% of CO_2_, the plate was promptly read as 20 repetitions, one every 60 s using Enspire (Perkin Elmer), at a controlled temperature of 37 °C. [3].

Positive and negative controls were previously prepared and incubated at 37 °C, 5% CO_2_ for 90 min before being added to the microplate.

The same procedure was also performed by placing the resins in contact with the L929 in plasma. In this case, controls were prepared with plasma in place of culture media.

For each well, the change in the wavelength peak of the various readings compared to the baseline was defined as response (*R*), calculated as follows:

(2) $$R\left(t\right)=S\left(t\right)-S^{baseline}(tb)$$

where *S*(*t*) is the final reading at time *t* and *S^baseline^*(*tb*) is the baseline at time *tb*, which was the same for all final readings of the assay. The responses (*R*) obtained are reported as mean ± SEM and displayed on a graph.

In addition, a further analysis of the data was performed by subtracting the average of the medium values from the response values relating to DMSO 0.2%, SDS 0.1%, Resin 1, and Resin 2 in culture medium. Values thus calculated, defined as “subtracted values” of the blank—where blank is represented by the negative control of cells in contact with culture medium—allowed us to view the real trend of the response of cells to the compounds tested.

### 2.8. Live and Dead Staining

To better show the cell morphology and confirm the data obtained by ELF, Live and Dead staining was performed using a LIVE/DEAD Cell viability/cytotoxicity assay kit (ThermoFisher Scientific, Eugene, OR, USA). Briefly, cells were stained with 500 μL of the LIVE/DEAD solution containing 2 μM calcein AM and 4 μM Ethidium Homodimer and incubated for 20 min at 37 °C/5% CO_2_. After staining, the wells were rinsed in PBS and immediately analyzed using an Evos optical invert microscope (Life Technologies, Carlsbad, CA, USA. Images were acquired at 10× magnification. Live and Dead staining marks the live cells in green and the dead ones in red.

### 2.9. Evaluation of Cytotoxicity in an Indirect Cell–Resin Contact with Customized Well Inserts Model by ELF

Once the response of L929s to direct contact with resins was evaluated using label-free technology, the experiment was repeated using indirect contact, via the designed and 3D-printed well inserts. They were introduced for all setting conditions: specimens and positive and negative controls obtained with both culture medium and human plasma.

After two hours of incubation at 37 °C of the test plate, the baseline reading was taken for 5 repetitions, one every 60 s. Subsequently, the well inserts loaded with resins were placed in the microwell plate, as well as 0.1% SDS and 0.2% DMSO controls, and after 24 h of incubation at 37 °C and 5% of CO_2_, 20 measurements every 60 s were performed in Enspire (Perkin Elmer), at a controlled temperature of 37 °C, and with the analysis of the data obtained as described above.

### 2.10. Statistical Analysis

To evaluate the robustness of the assay in terms of reliability and repeatability of the data, the calculation of the Z’-Factor was introduced, which is a parameter that takes into account the means and standard deviations of the responses of the positive and negative controls. Z’-Factor is calculated with the following formula:

$$Z’-Factor=1-\frac{\left(3\times Standard\;Deviation\;Maximum\;Signal\right)+\left(3\times Standard\;Deviation\;Minimum\;Signal\right)}{\left|(Mean\;Max.\;Signal-Mean\;Min.\;Signal)\right|}$$

In our case, the Maximum Signal is represented by the responses induced by the medium, since it provides a response with increasing values (positive and/or negative) compared to those relating to 0.1%. SDS Conversely, the Minimum Signal is represented by the 0.1% SDS, since it induces a response with decreasing values (positive and/or negative) when compared with those of 0.2% DMSO. In order to state that the assay is reliable and therefore reproducible, the Z’-Factor values must be between 0.5 and 1 (absolute value). Z’-Factor values lower than 0.5 indicate a poor reproducibility and therefore a low robustness of the assay.

The *t*-test (Excel, Microsoft^®^ Office 2016) was performed for the statistical analysis of the data [11]. The data were considered statistically significant if *p*-value (*p*) < 0.05. For greater robustness and reproducibility of the data, all experiments were performed in triplicate.

## 3. Results

### 3.1. Resin Cytotoxicity Evaluated by MTT Assay

**Figure 4:**
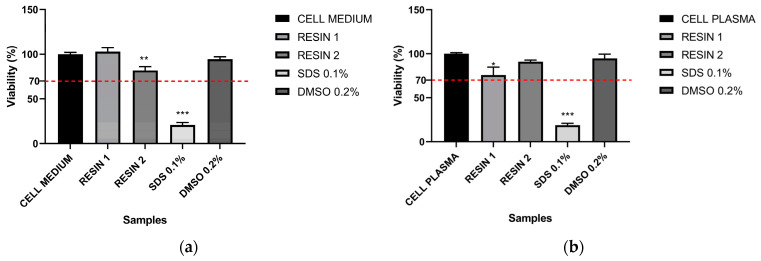
MTT assay of resins. Cell viability of L929 cells after exposure to resin extracts. (**a**): Resin extracts were obtained in culture medium. (**b**): Resin extracts were obtained in plasma. SDS 0.1% and DMSO 0.2% were used as positive and negative control, respectively. *p* value <0.05 (*) and <0.01 (**) and <0.001 (***) were considered statistically significant.

MTT assay was perform to evaluate the biocompatibility of Resins 1 and 2. As shown in Figure 4a (left panel), when cell culture medium was used to extract preparation, no significative toxicity was detected in stimulated cells, in a similar manner to the negative control (cell medium) and DMSO-treated cells. Cells treated with Resin 1 and Resin 2 showed a viability rate of 102.93 ± 4.35% and 81.77 ± 4.23 respectively. On the contrary, when SDS was added to the culture, a viability rate of only 20.8 ± 2.7 was registered, confirming the statistically relevant potential of SDS (*p*-value = 0.0000001).

Similar results were obtained when the extract was performed in plasma. No significative cytotoxicity was detected when cells were stimulated by resin extracts, as reported in Figure 4b (right panel). Stimulated cells showed a viability rate of 175.8 ± 8.9% for Resin 1 and 90.92 ± 1.83 for Resin 2, compared to the negative control. As expected, DMSO was inert, whereas SDS strongly affected the viability of the cells (18.85 ± 2.03%), in a statistically significant manner (*p* value = and 0.000004).

### 3.2. Resin Cytotoxicity Evaluated by Direct Contact

**Figure 5:**
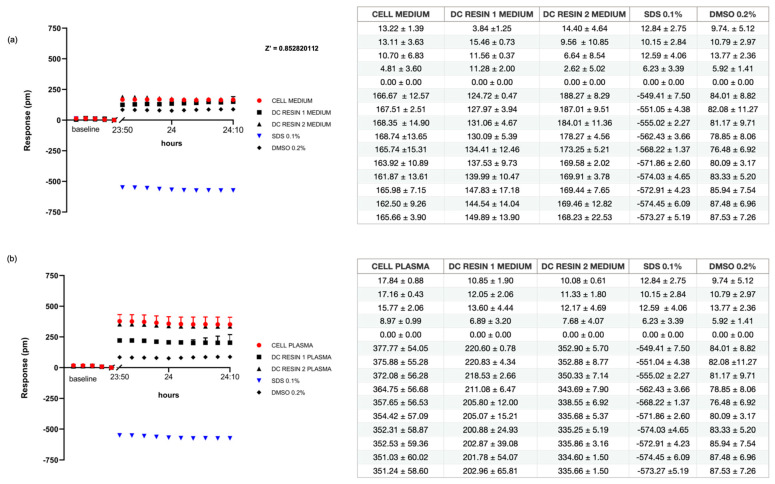
Label-free assay following direct contact in culture medium and plasma. Graphs display the response of L929 cells in direct contact with Resin 1 and Resin 2 in culture medium (**a**) and plasma (**b**). Samples are compared against negative internal controls represented by culture medium or plasma, 0.2% DMSO, and against positive control (SDS 0.1%). Z’ Factor > 0.5. In the tables, the mean ± SD of each sample is reported. The first five points represent the baseline. The other ones are the mean of each sample. Average is the mean of six points. Referring to the cell medium negative control, *p*-values obtained were Resin 1, 0.032; Resin 2, 0.025; SDS 0.1%, 0.0000012; and DMSO 0.2%, 0.1. Referring to cell plasma negative control, *p*-values were Resin 1, 0.0029; Resin 2, 0.017; SDS 0.1%, 0.00003; and DMSO 0.2%, 0.9.

Direct contact of the resins on fibroblasts was investigated using ELF and results are reported in Figure 5a. The effects observed with introduction of Resin 1 and Resin 2 were compared with two types of negative controls: cells in culture medium and cells in 0.2% DMSO (as required by the technology), and positive controls obtained by introduction of 0.1% SDS.

After 24 h, both resins showed a similar trend compared to the negative control represented by the medium, with a response of 169 ± 14 pm, while the 0.2% DMSO-treated sample presented a slight positive response of 80 ± 3 pm. On the other hand, the positive control displayed a negative trend (−549 ± 4 pm) at the first readout, which was confirmed at t = 24 h (−571 ± 2 pm).

The measurements performed on cells in direct contact with resins appear to be comparable to those carried out on the negative control, confirming the data obtained by MTT assay, i.e., that no cytotoxicity effects occurred.

Then, we analyzed the effect of resins when medium was replaced by human plasma.

In Figure 5b and in the relative table, we observe a positive response of 205 ± 15 pm and 335 ± 5 pm, respectively, for Resins 1 and 2 in the presence of plasma. Similarly, the negative controls, obtained from culturing cells with plasma only, showed a positive response of 352 ± 59 pm, while analysis of 0.2% DMSO samples revealed a signal of 80 ± 3 pm. Conversely, the analysis of 0.1% SDS samples showed strongly negative values, since the first repetition (−572 ± 4 pm), reaching −571 ± 2 pm at 24 h. The direct contact with the resins in plasma appears to be comparable to the negative control, suggesting that no cytotoxicity effects occurred, but we were able to observe an increase in ∆pm of 130 pm in Resin 2 compared to Resin 1. The statistical result of the Z’-Factor exceeded the threshold value of 0.5, reaching 0.85, indicating the test results were robust and reproducible.

**Figure 6:**
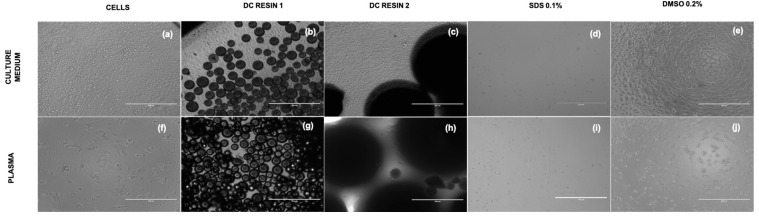
Images of L929, cultured both in medium and plasma in transmitted light. The figures report L929 cells cultured for 24 h with controls and in direct contact with Resin 1 and Resin 2 in culture medium (**a**–**e**) and plasma (**f**–**j**). The pictures were acquired at magnification of 10×, scale bar = 400 µm.

Microscope observation showed a physiological morphology of L929 in culture media (Figure 6a–e) and an increased cell size in all samples treated with plasma (Figure 6f–j), confirming the different response observed by ELF. Of course, cells incubated with 0.2% SDS showed damaged cells, as expected (Figure 6d,i).

**Figure 7:**
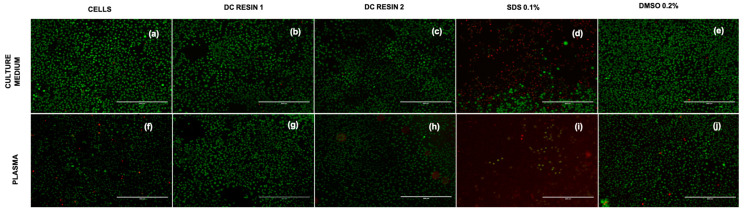
Images of L929, cultured both in medium and plasma stained with Live/Dead. The figures report L929 cells cultured for 24 h with controls and in direct contact with Resin 1 and Resin 2 in culture medium (**a**–**e**) and plasma (**f**–**j**). The pictures were acquired at magnification of 10×, scale bar = 400 µm.

Furthermore, to better investigate the morphology of the cells in the different condition, Live and Dead staining was performed (Figure 7a–j). Staining confirms the cell viability both in culture medium and plasma and in direct contact with the resins (Figure 7a–c,f–h). On the contrary, SDS strongly affected cell viability, as indicated by the great number of red cells (Figure 7d,i).

### 3.3. Resin Cytotoxicity Evaluated by Indirect Contact That Simulate the Clinical Use

To better simulate the clinical contact of the resins with patients, we introduced an indirect contact by using customized well inserts, obtained with the Stratasys^®^-Eden 260V printer, which have five pores of 530 µm (diameter) in the lower surface to allow exchange between the resin and the medium in contact with cells.

**Figure 8:**
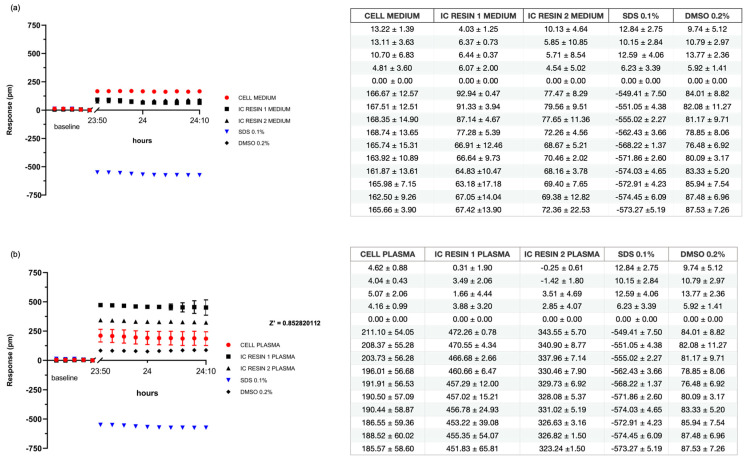
Label-free assay following indirect contact in culture medium and plasma. Graphs display the response of L929 cells in indirect contact (IC) with Resin 1 and Resin 2 in culture medium (**a**) or plasma (**b**). Samples are compared against negative controls represented by culture medium or plasma, 0.2% DMSO, and against positive whole controls (SDS 0.1%). Z’ Factor > 0.5. In the tables, the mean ± SD of each sample is reported. The first five points represent the baseline. The other ones are the mean of each sample. Average is the mean of six values. Referring to cell medium negative control, *p*-values obtained were Resin 1, 0.02: Resin 2, 0.019; SDS 0.1%, 0.0000031; and DMSO 0.2%, 0.098. Referring to cell plasma negative control, *p*-values were Resin 1, 0.00000036; Resin 2, 0.000015; SDS 0.1%, 0.00000002; and DMSO 0.2%, 0.12.

As shown in Figure 8a and in the relative table, after 24 h of incubation of samples in culture media, Resins 1 and 2 had trends similar to that of the control in the 0.2% DMSO (76 ± 7 pm), with a response of 67 ± 12 pm and 69 ± 5 pm, respectively, which is also comparable to the culture media, which had a positive response of 165 ± 12 pm. On the other hand, SDS 0.1% (positive control) had a negative trend (−568 ± 1 pm), which was confirmed at t = 24 h (−571 ± 3 pm) since the first measurement.

After the analysis of the response of L929 cells to indirect contact via well inserts with the resins in the medium, we then evaluated the effect in plasma.

After 24 h, Resins 1 and 2 showed response of 457 ± 15 pm and 328 ± 5 pm, respectively, with a similar trend to L929 in human plasma, and in 0.2% DMSO, showed values of 190 ± 57 pm and 80 ± 3 pm, respectively.

On the contrary, SDS 0.1% (positive control) reported negative values, right from the first repeat (−568 ± 1 pm), reaching −571 ± 3 pm at 24 h. The statistical result of the Z’-Factor exceeded the threshold value of 0.5, reaching 0.85, indicating the test results were robust and reproducible.

Although trends were similar in both negative controls, addition of resins, particularly Resin 1, showed a slight increase in peaks in a statistically significant manner compared to control samples (*p* value < 0.01). Through the tests performed with ELF, therefore, it was possible to analyze details of the cellular response that were otherwise not appreciable.

**Figure 9:**
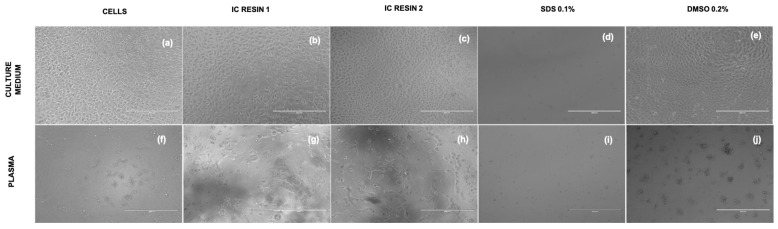
Images of L929, cultured both in medium and plasma in transmitted light. The figures report L929 cells cultured for 24 h with controls and in indirect contact with Resin 1 and Resin 2 in culture medium (**a**–**e**) and plasma (**f**–**j**). The pictures were acquired at magnification of 10×, scale bar = 400 µm.

Microscope analysis showed a physiological morphology of L929s in culture media (Figure 9a–e) and an increased cell size in all samples treated with plasma (Figure 9f–j), except for samples incubated with 0.2% SDS (Figure 9d,i).

**Figure 10:**
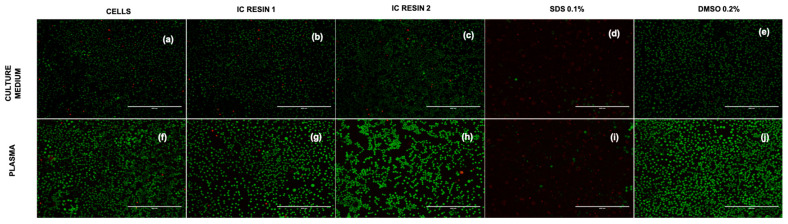
Images of L929, cultured both in medium and plasma stained with Live/Dead. The figures report L929 cells cultured for 24 h with controls and in indirect contact with Resin 1 and Resin 2 in culture medium (**a**–**e**) and plasma (**f**–**j**). The pictures were acquired at magnification of 10×, scale bar = 400 µm.

Furthermore, to better investigate the morphology and viability of the cells in the different conditions, Live and Dead staining was performed (Figure 10a–e). Staining confirms the cell viability both in culture medium and plasma and in indirect contact with the resins (green cells in Figure 10a–c,f–h). On the contrary, SDS strongly affected cell viability, as indicated by the great number of red cells (dead cells) (Figure 10d,i).

By microscope observation only, no differences were appreciated between the negative control and resin or between the two types of resins, while ELF was able to emphasize a difference between samples.

## 4. Discussion

The development of new approaches in the biocompatibility field that are able to obtain accurate, predictive, and rapid information regarding the material/cell interaction has become necessary in the framework of biomedical regulation [9,12].

The UNI EN ISO 10993-1:2018 standard [9] aims to define the minimum requirements for assessing biocompatibility on medical devices (MDs), providing protocols according to the type and duration of contact with patients. However, it often involves the use of in vivo tests on animals (mice, rabbits, pigs), which are, in addition to being very expensive and time-consuming, subject to important ethical implications. Furthermore, with the evolution of technologies and the emergence of new materials, we tried to respond to market needs, looking for a new technology that could act as a link between the legislation and the “new advancing”.

The aim of the study was to translate a latest generation technology, ELF, which is already used for drug discovery studies [13,14] or cell pathway evaluation [1], into the context of biocompatibility tests.

In a previous work published by our group [3], ELF technology was applied to the study of biocompatibility of methacrylate resin commonly employed for cranioplasty surgery. In order to mimic in vitro the intended use of the MD, the test was performed on osteoblasts, HUVEC, and iPS-derived neurons, which are all cell types that normally interact with resin in the in vivo microenvironment. In that study, ELF was able to evaluate the cellular response to methacrylate resin by comparing its profile with controls, and the non-toxic effect of the material was confirmed [3]. Based on our previous experience, in this study, we aimed to reach a new goal, consisting of the application of ELF to evaluate the cell/material interaction and the cell response upon contact. We focused the attention on commercially available ion-exchange and macroporous polymeric resins, which are placed inside the blood filters to sequester molecules from the bloodstream [6], representing a relevant group of MDs and the only life-saving therapy for patients with kidney failure [15].

We applied ELF to investigate biological effects in a relevant microenvironment mimicking clinical use, with the aim of providing a detailed picture of what happens in the body when resins come in contact during dialysis.

In order to maintain a comparison with cytotoxicity tests (according to ISO 10993-5) and to better appreciate any additional information on the cellular response, the experiments were performed using the murine fibroblast cell line L929 [1,9]. For the same reason, the cell–material contact was maintained for 24 h as reported in the MTT protocol.

The binomial ELF/Enspire (technology/tool) can provide more information about cell response compared to the standard assays. Due to the sensitivity of the instrument used for the readings and the perceptiveness of the plate, even minimal changes in cell morphology can be detected, which would otherwise not be appreciable with other techniques able to detect live or dead cells. Moreover, the trend of the cellular response over time after the addition of the compound can also be monitored, without focusing on a single time point, but with a 360-degree overview. Since resins are normally used inside blood filters to sequester molecules from the bloodstream, we considered it relevant to analyze the cellular response in a microenvironment that mimics the clinical scenario. We finally identified human plasma, as the element able to mimic the interaction of blood with resin, to investigate biological effects on cells considering that interaction of materials in the presence of plasma might elicit a different effect compared to culture media.

ELF tests performed in culture medium (which was our negative control), exploiting the direct cell–material contact, confirmed the biocompatibility previously evaluated by MTT assay, and confirmed the viability of cells as reported by Live and Dead assay. Curiously, with the addition of plasma, we observed a different biological behavior of cells. L929 incubated with the resins showed a modification in the distribution of the cell mass; in particular, in the presence of Resin 2, cells showed a lower P-DMR than cells incubated with Resin 1 or with plasma alone. This may be due to the activation of two different cellular pathways [16] depending on contact with Resin 1 or Resin 2.

To better mimic the clinical scenario, we further introduced an indirect assay by using customized well inserts suitable for the dimensions of the wells of the label-free plate, internally produced with a 3D printer, since there are no similar products on the market. Indirect contact allows the monitoring over time of biological effects caused by resin alone or by a compound interacting with culture media or plasma. Moreover, any artefact, such as interference in readings due to interaction of microspheres with the sensor and eventual damage of cells caused by direct contact, can be excluded. Thus, the test performed by combining an innovative technology such as ELF and the development of a customized support allowed us to detect the cell response to the presence of the resins, improving the performance of the technology itself and providing molecular details that would otherwise not be found.

In detail, the trends of Resins 1 and 2 in plasma were similar to those of the negative controls in the experimental setting with or without plasma indirect/direct contact. In particular, Resin 1 in the presence of plasma exerted a biological effect that was different from that of Resin 2, causing a slight increase in the response in a statistically significant manner compared to the control samples in the presence of plasma in indirect contact. However, a slight DMR was also observed in direct contact in the presence of plasma. Resin 2 is an anion-exchange resin that works by changing ions with blood. Our model suggests that Resin 2 may interact with plasma in a unique manner, leading to an increase in cell mass that is not observed in culture media.

This detailed cellular response would not otherwise have been visible, without the sensitivity of the ELF technology. Further investigation is needed to evaluate which pathway is activated in the cell–material–plasma contact.

Thanks to the label-free technology, which was applied here in the biocompatibility field, we better appreciated the specific sequential cellular response of L929, showing peculiar trends that were suggestive of the activation of possible intracellular pathways.

## 5. Conclusions

In conclusion, ELF could represent a good candidate technique to obtain a specific “biological material profile” and to determine the similarities among materials. In the biocompatibility field, materials or medical devices are defined equal if all procedures and phases involved in their manufacturing process are the same (ISO10993-1:2018). Given the high sensitivity of ELF, it could be considered as an approach to compare two batches of the same material or to evaluate the equivalent biocompatibility of materials defined as equal.

Today, ELF can represent an important source of additional information regarding the response of the human organism to a life-saving therapy such as dialysis, which is currently the only alternative to organ transplantation. In the future, this technology will be able to be further developed and applied to many other cellular therapies by providing data that would otherwise remain unknown.

## Data Availability

Data available on request due to privacy restrictions. The data presented in this study are available on request from the corresponding author. The data are not publicly available due to privacy restrictions.
